# Modeling and Deploying IoT-Aware Business Process Applications in Sensor Networks

**DOI:** 10.3390/s19010111

**Published:** 2018-12-30

**Authors:** Yongyang Cheng, Shuai Zhao, Bo Cheng, Xiwei Chen, Junliang Chen

**Affiliations:** 1 State Key Laboratory of Networking and Switching Technology, Beijing University of Posts and Telecommunications, Beijing 100876, China; chengbo@bupt.edu.cn (B.C.); chjl@bupt.edu.cn (J.C.); 2 School of Public Health, Indiana University Bloomington, Bloomington, IN 47405, USA; xiwechen@indiana.edu

**Keywords:** Internet of Things, business process modeling, deploying algorithm, sensor networks

## Abstract

The concept of the Internet of Things (IoT) is an important part of the next generation of information. Wireless sensor networks are composed of independent distributed smart sensor nodes and gateways. These discrete sensors constantly gather external physical information, such as temperature, sound, and vibration. Owing to the diversity of sensor devices and the complexity of the sensor sensing environment, the direct modeling of an IoT-aware business process application is particularly difficult. In addition, how to effectively deploy those designed applications to discrete servers in the heterogeneous sensor networks is also a pressing problem. In this paper, we propose a resource-oriented modeling approach and a dynamic consistent hashing (DCH)-based deploying algorithm to solve the above problems. Initially, we extended the graphic and machine-readable model of Business Process Model Notation (BPMN) 2.0 specification, making it able to support the direct modeling of an IoT-aware business process application. Furthermore, we proposed the DCH-based deploying algorithm to solve the problem of dynamic load balancing and access efficiency in the distributed execution environment. Finally, we designed an actual extended BPMN plugin in Eclipse. The approach presented in this paper has been validated to be effective.

## 1. Introduction

The Internet of Things (IoT) connects everything to the Internet through numerous information sensing devices to realize intelligent identification and management. Various smart sensors are the fundamental building blocks for the creation of an IoT-aware business process application [1,2]. A business process management (BPM) system not only helps to improve the efficiency of collaboration among cross-sensor business processes, but also helps to invoke appropriate treatment measures before emergencies snowball into disasters (e.g., fire hazard, traffic jam, network failure, etc.) [3,4]. Because of the diversity of sensors and differences in their functions, the direct modeling of an IoT-aware business process application is particularly difficult [5]. Developers might need to spend a lot of energy on coding, resulting in ignoring the orchestration of business logic [6]. As we know, the basis of an IoT application before any business process automation is the creation of a business process model [7]. A model is composed of all kinds of business process tasks, which correspond to special function units implemented by services. A BPM system would benefit from directing modeling if typical sensor devices could take over responsibility as sensor resources for individual business process tasks [8,9]. Furthermore, for a large-scale modern IoT application, numerous smart sensors are discretely deployed in multiple monitoring areas in the distributed sensor networks [10]. These smart sensors not only generate massive sensing data, but also hope to get a response from the servers as soon as possible. Thus, the distributed storage is considered reasonable as a result of measuring the efficiency, security, and robustness. Then, how to deploy the designed IoT-aware business process applications to distributed servers in the heterogeneous sensor networks is a pressing issue.

In order to build our work on a scientific foundation, we have investigated most of the existing business process approaches (Business Process Model Notation (BPMN) 1.0, Business Process Execution Language (BPEL), and JBoss Process Definition Language (JPDL)). However, none of the existing modeling languages could meet our needs to directly model a sensor-aware business process application. Compared with other modeling languages, the biggest advantage of BPMN 2.0 is that it could define the execution semantics and format of the specification and use the standard primitives to describe the business process, ensuring that the same process gets the same execution results in different process engines. Besides, our development work is based on an open source project Java Business Process Management (JBPM) 6.1.0, which provides various capabilities that simplify and externalize business logic into reusable process resources and follows the BPMN 2.0 specification. Thus, BPMN 2.0 serves as the basis for the mapping work of business process resources from an IoT domain to a standard process task in this paper. Although it could provide theoretical support for the modeling and some strategies are proposed to solve the problem of efficient deploying, challenges still remain to be solved [11,12,13,14,15,16,17,18,19,20,21,22]. First of all, the role of sensor devices as a resource type of the business process could not be directly represented in BPMN 2.0 specification, which means that sensor devices could not be considered as potential executive parties in the automatic resolution phase. Furthermore, because of the discrete distribution and complex sensing environment, an efficient deploying algorithm considering both load balancing and access efficiency is urgently needed. However, to our best knowledge, most of the current work pays little attention to this problem. Finally, the development process for developers is still a complicated and time-consuming issue. They need not only to focus on the mapping of entity resources and the orchestration of business logic of an IoT-aware business process application, but also to have an excellent programming skill.

The main contributions of our work in this paper include the following:

- We extend the graphic model and machine-readable model consisting of the XML Schema Definition (XSD) specification of BPMN 2.0, which could formally describe the elements in an extended XML document, making it able to support the direct modeling of a sensor device.
- We propose a novel deploying algorithm based on dynamic consistent hashing (DCH) to solve the problem of dynamic load balancing and access efficiency in the heterogeneous sensor networks. On this basis, the overall performance has been significantly improved.
- We qualitatively and quantitatively analyze the proposed approach. In addition, the work presented in this paper has been implemented through an actual development system.

The rest of this paper is organized as follows: We discuss the related works and clearly point out the similarities and differences in Section 2. In Section 3, the formal modeling specifications of a smart sensor device, including both graphic and machine-readable models, are investigated. We propose a novel deploying algorithm to solve the problem of load balancing and access efficiency in Section 4. In Section 5, we discuss the feasibility of our proposed approach and evaluate it through a series of simulation experiments. On the basis of our proposed approach, we describe the details of implementation and discuss advantages and disadvantages of our developed IoT system in Section 6. An actual IoT application scenario based on our system in the forest protection is described in Section 7. Concluding remarks are made in Section 8.

## 2. Related Works

In this section, we mainly compare our proposed approach with other existing approaches. We will clearly point out the similarities and differences between their works.

### 2.1. Modeling for the IoT-aware Business Process Applications

Dave et al. [11] described a platform that integrated the built environment data with IoT sensors in a campus-wide, web-based system that provides information about energy usage, occupancy, and user comfort by integrating building information models and IoT devices through open messaging standards (0-MI and 0-DF) and Industry Foundation Classes (IFC) models. Rosa et al. [12] considered the construction of unions of multiple models (called merged models), as well as intersections (called digests). Merged models are intended for analysts who wish to create a model that subsumes a collection of process models, typically representing variants of the same underlying process, with the aim of replacing the variants with the merged model. Smirnov et al. [13] systematically developed and consolidated the use cases for business process model abstraction and presented a case study to illustrate the value of their proposed technique. Schulte et al. [14] conceptualized an architecture for an elastic BPM system and discussed existing work on scheduling, resource allocation, monitoring, decentralized coordination, and state management for elastic processes. Furthermore, they presented two representative elastic BPM systems that intended to counter these challenges. Meyer et al. [15] presented how the real-world services could be considered as a business process resource type. Rosa et al. [16] drew up a systematic inventory of approaches to modeling and provided a comparative evaluation thereof with the aim of identifying common and differentiating modeling features, providing criteria for selecting among multiple approaches, and identifying gaps in the state of the art.

### 2.2. Deploying Algorithms for the Discrete IoT Applications

Lin et al. [17] proposed a programming model for deploying IoT devices in respective potential sites under the constraints of limited capacity. Their ultimate goal was to reduce the installation cost. Senouci et al. [18] sought to address the problem of resource detection and healing in mobile Wireless Sensor Networks (WSNs). They discussed the main drawbacks of existing solutions and identified four key elements critical for ensuring effective coverage in mobile WSNs. Furthermore, they proposed a lightweight and comprehensive solution, called resources detection and healing (HEAL). Jiang et al. [19] proposed a depth adjustment algorithm based on connected tree (CTDA). In CTDA, the sink node is used as the first root node to start building a connected tree. Finally, the network can be organized as a forest to maintain network connectivity. Coverage overlaps between the parent node and the child node, which are then reduced within each sub-tree to optimize coverage. Kim et al. [20] proposed a new hash-based mapping algorithm, which reduces the redundant operations and uses only a few memory resources for the mobile storage device. Liang et al. [21] proposed a location-aware service deployment algorithm based on K-means for the cloudlets. Simply speaking, the proposed algorithm divides mobile devices into a number of device clusters according to the geographical location of mobile devices and then deploys service instances onto the edge cloud servers nearest to the centers of device clusters. Hwang et al. [22] developed a performance model, which defines manufacturing processes and performance indicator formulas.

### 2.3. Summary of Related Works

The above-mentioned approaches are generally still in their infancies. The integrating model in the work of [11] is human-oriented and the modeling is complicated because of the lack of theoretical support for direct mapping from physical sensor entities to standard business process resource tasks. The merging operator in the work of [12] relies on a mapping between the changeless nodes of input models, which could not adapt to the dynamic IoT execution environment. Although the work of [13] proposed an approach to cooperate with end users, it lacks of a clear understanding of what distinguishes these techniques, and how they address real world use cases has not yet been established. The work of [14] should take into account more business and application driven research questions and analyze the applicability of elastic processes in different application domains. Although the work of [15] also extended BPMN 2.0 standard at the level of a meta-model, this extension was based on the lane. In contrast to these works, we extended the standard at the level of activity, which could directly inherit the associations of ResourceRole. Besides, we decoupled the internal connection between sensor devices through a special class, which supports the dynamic assignment of entity resources. The work of [16] requires more complex indirect mappings that are needed when sensor-associated elements are used. The work of [17] ignores the load balancing of distributed servers and the access efficiency of deployed IoT-aware business process applications. However, the computation complexity of HEAL in the work of [18] is O(v^2^); they should reduce the time complexity and investigate the interaction between HEAL and the network layer. The hierarchical strategy in the work of [19] is used to adjust the distance between the parent node and the child node to reduce node movement. However, the energy consumption of CTDA is less than that of connected dominating set (CDA), particularly in a distributed environment. Similar to the work of [20], we also built our work on the basis of a hashing algorithm. However, the number to conduct the hash operation in this approach relies on servers, which could not adapt to the dynamic execution environment. The work of [21] focused only on network latency for reducing the response time. A service consists of computation, communication, and I/O. Thus, they should take these factors into account for designing an advanced service deployment. The automation in the work of [22] is limited. In order to develop and manage an IoT application, users have to do large amounts of coding work, including the configuring of parameters, compiling of XML files, and validating of the model. This not only takes lots of time and energy, but also easily leads to ignoring the orchestration of business logic.

Besides the theoretical methods, in order to automate the approach presented in this paper, we have developed an actual system for developers to manage the full lifecycle of the IoT-aware business process applications. Our work is based on an open source project JBPM 6.1.0 [23], which is an Eclipse-based editor to support the graphical creation of business processes. However, the existing functions could not meet our needs to directly model and efficiently deploy business process applications. Therefore, we extended them and developed a series of custom buttons.

## 3. The Resource-Oriented Modeling Specification

In this section, we will propose a new resource-oriented modeling specification as an extension to BPMN 2.0 specification. When integrating the paradigm of a sensor device on the business process layer, we faced two main challenges. First, the role of sensor devices as a business process resource could not be directly represented in the basic element library. Second, it could not directly parse the sensor associated service in the process execution engine, which is not known in XML format to the BPM environment. However, the existing BPMN 2.0 specification could not fully consider these two above issues on different levels concurrently in the same business process model. Thus, we extended both the graphic model and the machine-readable model. We briefly introduced this part of work and published it at the 20th International Conference on Model Driven Engineering Languages and Systems (MODELS) [24]. However, because of the limitation of paper length and work schedule, we only analyzed the feasibility in theory, lacking implementation details. To make it easier for readers to understand the advancements of our work, we have clearly refined them, including the popular interface technologies to access the IoT service, the common data format to encapsulate heterogeneous sensor data, and the operation to specify the operation (control flow or message flow) that will be invoked by the sensor task. In addition, we integrated two classes into one class handler to optimize code structure and improve execution efficiency.

### 3.1. The Graphic Model

In order to directly model a sensor as a business process resource in the basic element library, we need to consider the following three aspects: *Sensor Device*, *Sensor Service*, and *Handler*. *Sensor Device* determines the entity type of a sensor. For example, it is either a temperature sensor, a light sensor, or a pressure sensor. In general, the essential function of a sensor is to perceive its surrounding environment and send data to the information center. *Sensor Service* defines the function that could be provided by a specific sensor. However, the service is not directly visible as a part of the business process model, but is included in the implementation and operations, which could be implemented in *Handler*. Implementation specifies the technology that is used to access the service. Valid values are “##undefined” for leaving implementation technology open, “##Restful” for the restful technology, or an Uniform Resource Identifier (URL) identification. Restful is the default technology. Operation specifies the operation that will be invoked by the sensor.

**Figure 1:**
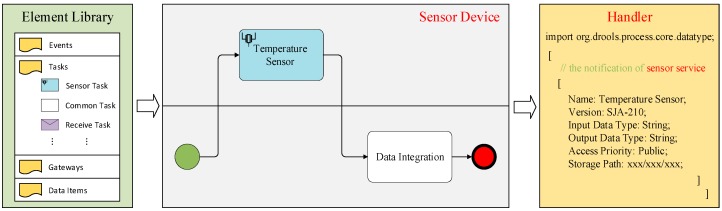
A business process model of the temperature perception associated application.

In BPMN 2.0 specification, *Activity* is work that is performed within a business process and could directly inherit the associations of *ResourceRole*, which inherits the attributes of *BaseElement*. An *Activity* class is an abstract element, which represents the point in a process flow where work could be performed. In order to explicitly distinguish sensor devices from the traditional business process performers and bind sensor services to the graphical element through an interface component, we design a characteristic icon, called *Sensor Task*, at the level of *Activity*. The designed task *Sensor Task* shares the same shape as the common task, which is a rectangle that has rounded corners. However, there is a graphical sensor in the upper left corner of the shape that indicates that the task is a *Sensor Task*. We provide the interfaces’ implementation and operation in attributes for developers to bind services and define the parameters of a specific sensor. In order to illustrate the use of the graphical model, Figure 1 illustrates a business process model of the temperature perception associated application. The left of Figure 1 shows the basic element library, where developers create this business process model through dragging and dropping the corresponding process tasks. The middle of Figure 1 shows the designed business process model, which contains a regular line and an IoT-aware sensor line, namely *Sensor Device*, that could expose its service through a restful interface. The right of Figure 1 illustrates the configuration of relevant parameters, including sensor name, version, input/output data type, access priority, and storage path.

### 3.2. The Machine-Readable Model

Based on the extension of the graphic model, we could directly create a sensor-aware business process through dragging and dropping the corresponding icons from the element library. However, there is another problem to be solved. That is, although BPMN 2.0 specification already supports the allocation of resources at the *Activity* level [25,26], it could not directly parse the sensor associated service in the business process execution engine, which is not known in XML format in the BPM environment. Thus, we also extend the machine-readable model.

To address the above issue, we initially introduced a subclass to the *Activity* class and directly brought the resource allocation of entity devices to the *Activity* level. This class is *Sensor Device* and inherits the model associations and attributes of the *Activity* class. By configuring the relevant parameters, the sensor information will be passed to the process execution phase in a generated XML document. In this case, an available *IoT Device* class could be found by the process execution engine at run-time. However, the definition of a *Sensor Device* class is abstract, because it could only define the types of resources, without detailing how to reference the entity resource. Thus, we design an *Interface* class to implement the abstract class *Sensor Device*. The *Interface* class inherits the model association and attributes of *BaseElement* and leaves interfaces for the *Sensor Service* class, which will be introduced next. All process elements contain IDs within the BPMN XML schema definition (XSD) and references to elements are expressed via these IDs. The XSD type is the traditional mechanism for referencing by IDs, however it can only reference an element within the same file. The BPMN XSD supports referencing by ID and utilizing QNames. A QName consists of two parts: an optional namespace prefix and a local part. When used to reference a BPMN element, the local part is expected to be the ID of the process element. Table 1 presents the additional attributes and model associations of the *Interface* class and the corresponding XML schema definition (XSD) specification is described in Table 2.

**Figure 2:**
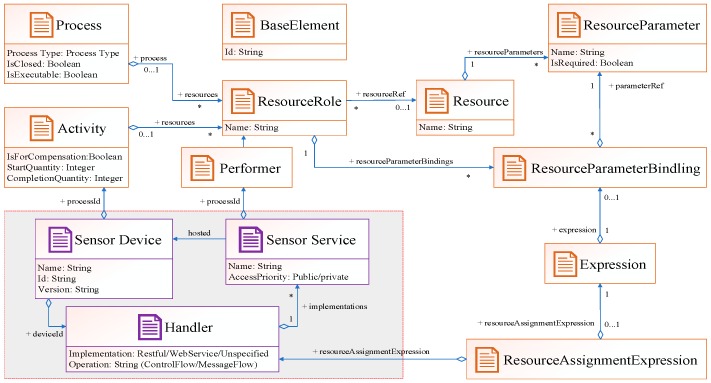
Sensor-aware specific class extension of the XML schema definition (XSD) specification of Business Process Model Notation (BPMN) 2.0.

As we know, the essential function of a sensor is to perceive its surrounding environment and send data to the information center. However, every sensor has its specific function and data format. For example, the temperature sensor gathers text-format data, the infrared thermal imaging sensor gathers image-format data, and the monitor sensor gathers video-format data. In order to better parse them in the process engine, we encapsulate the raw data in a unified message format, which has been introduced in our other work [27]. Besides the data format, how to directly access these sensor services in the machine-readable model is another problem. To address this issue, we designed a *Handler* class consisting of implementations and operations. Implementation specifies the technology that is used to locate a sensor service. Valid values are “##unspecified” for leaving the implementation technology open, “##Restful” for the restful technology, or a URL identification. Because of the widespread application of restful technology in sensor networks, restful is the default technology. Operation specifies the operation that will be invoked by the sensor task. There are two common situations: the first situation is directly invoking a new task in the control flow and the second one is indirectly invoking a new task by *SendTask* in the message flow. Consequently, a sensor resource could be defined and mapped from an entity domain to a machine-readable model, without being known previously to the BPM environment. Figure 2 illustrates the extension of the XSD specification in a machine-readable model. The elements in white areas are existing roles in BPMN 2.0 specification. The elements in gray areas (sensor device, sensor service, and handler) are new roles to extend the functions of process engine.

## 4. A DCH-Based Deploying Algorithm in the Heterogeneous Sensor Networks

### 4.1. Basic Deploying Algorithm

**Figure 3:**
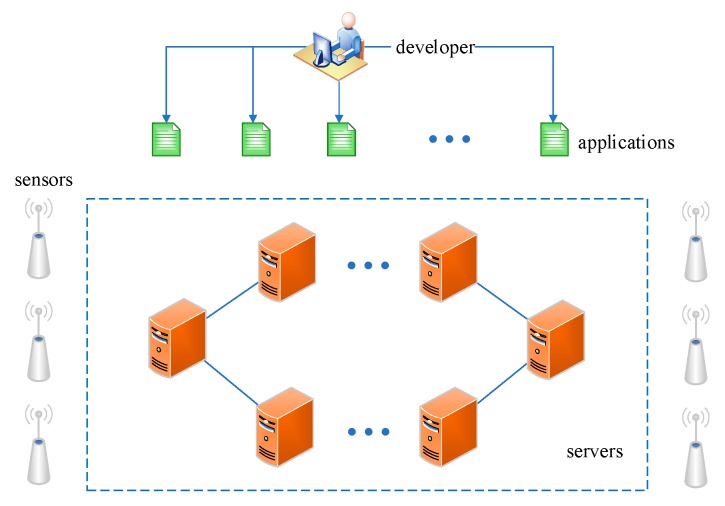
A distributed execution environment.

In the previous section, we introduced the formal modeling method for the IoT-aware business process application in heterogeneous sensor networks. As we know, a complete lifecycle of a sensor-aware business process application usually includes the following parts: modeling, deploying, and running. From the perspective of application execution, modeling is the design phase, where the developer could create the business processes. Because of the complexity of the execution environment, the business processes are usually cross-sensor collaborative processes. In order to get the goal of distributed execution, we should decouple them into fine-grained process fragments after the design phase. However, these decoupled process fragments are the basis for further deployment. We discuss the deploying algorithms based on the results during the modeling phase. For example, the number of process lanes determines the process fragments to be deployed; the process name and designing time would be used to conduct the hash operation; and the access efficiency is directly affected by the priority-based storage structure, which is just designed during the modeling phase. Thus, modeling and deploying are two closely related parts and are discussed together in this paper. In a large-scale modern IoT scenario, numerous servers are discretely placed to store the massive sensor data and applications developed by different developers to balance the system load, which is illustrated in Figure 3. Thus, besides the phase of modeling, how to deploy those designed IoT applications to discrete servers to ensure load balancing and access efficiency, especially in heterogeneous sensor networks, is another problem to be solved.

There are two common deploying approaches to address this issue [28,29,30,31]. The first one is sequence-based storage. We assume that there are n available servers and m designed applications in an IoT scenario, where n << m. All servers are assigned constants from 1 to n at initialization time. When deploying applications to these servers, developers deploy them based on a loop of the assigned constants of the servers from 1 to n. A pointer is set to mark the next available server. The advantage of this sequence-based approach is that the logic and implementation operation are simple. However, the shortcoming of it is a lack of flexibility. If we need to access or modify a deployed application, we have to go through every application of all the servers.

**Figure 4:**
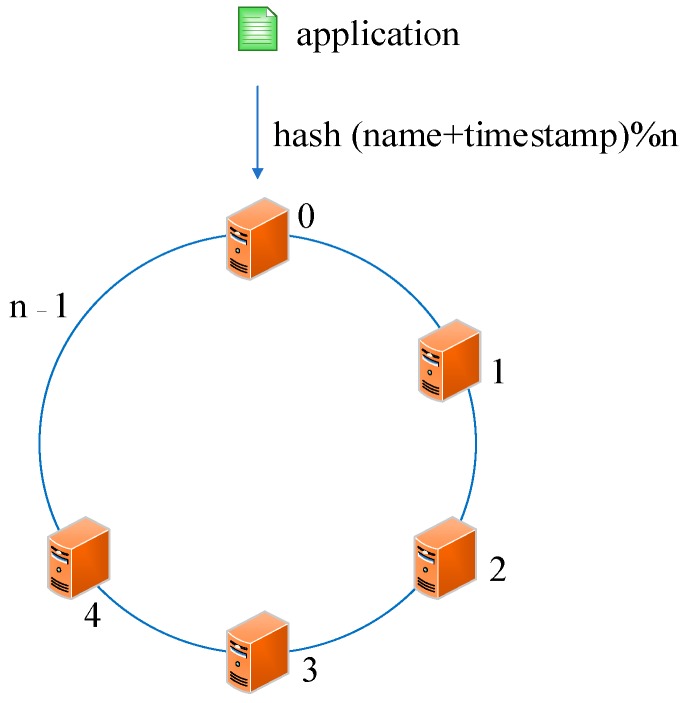
A sequence-based deploying approach.

The second approach is hash-based storage. Similar to the first sequence-based one, all servers are assigned constants from 1 to n at initialization time. In contrast, however, a custom function *hash ()* is designed to map the values of the applications to constants. To distinguish these applications, we define them by name and timestamp. Because the timestamp is not repeated, the constants are different after the operation of *hash (name + timestamp)*. We defined the symbol “%” as the operation to obtain a remainder in this paper. When deploying applications to the servers, we initially calculated the remainder i of the function *hash (name + timestamp)%n*, where i varies from 0 to n − 1. Then, we deployed the corresponding application to the server whose remainder is i. Through this approach, we can immediately get the number of the server that stores the required application when we need to access or modify a deployed application. Figure 4 illustrates this approach. Although this approach is more flexible than the first one, it still has two problems that cannot be ignored: (1) If a server becomes unavailable, the function must be turned to *hash (name + timestamp)%(n* − 1*)*, which means that a massive cache becomes invalid or numerous applications need to be transferred when we need to access or modify a deployed application. Similarly, If we add a server into the sensor networks, the function is turned to hash *(name + timestamp)%(n* + 1*)* and we are faced with the same problem. (2) In general, separate chaining is used to address the hash collision. Applications are stored on the same server in the form of a linked list if their remainders are the same value after the hash operation. However, this linking is based on the sequence. If we need to access or modify an application, we have to go through every application on this server.

**Figure 5:**
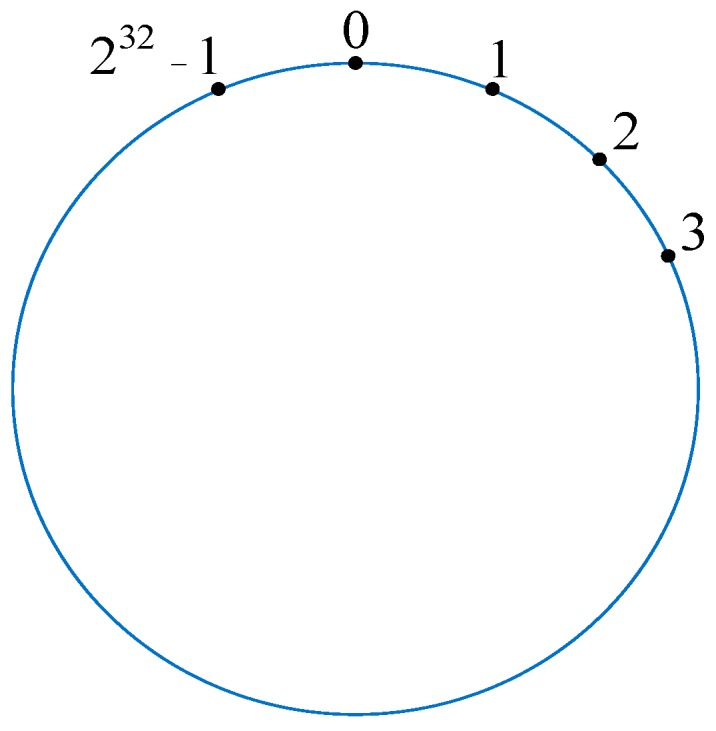
The designed hash circle.

To address the above problems, we proposed the dynamic consistent hashing (DCH)-based deploying algorithm. Similar to the traditional hash-based approach, we also need a number to conduct the hash operation. In contrast, however, this number is a constant 2^32^ instead of the number of server. The reasons for us choosing 2^32^ as the constant to conduct the hash circle are as follows: (1) Because it is a world of 0’s and 1’s in the computer, it is easy to distribute and balance the data with 2^m^ (m is a constant) when partitioning. (2) The IP address of the server is 32 bits and we use it to conduct the operation *hash()%(2^m^)*, thus we set the value of m to 32. We consider that there is a virtual circle consisting of 2^32^ nodes, which are assigned identifiers from the number 0 to 2^32^ − 1. We name and illustrate this virtual circle hash circle in Figure 5.

**Figure 6:**
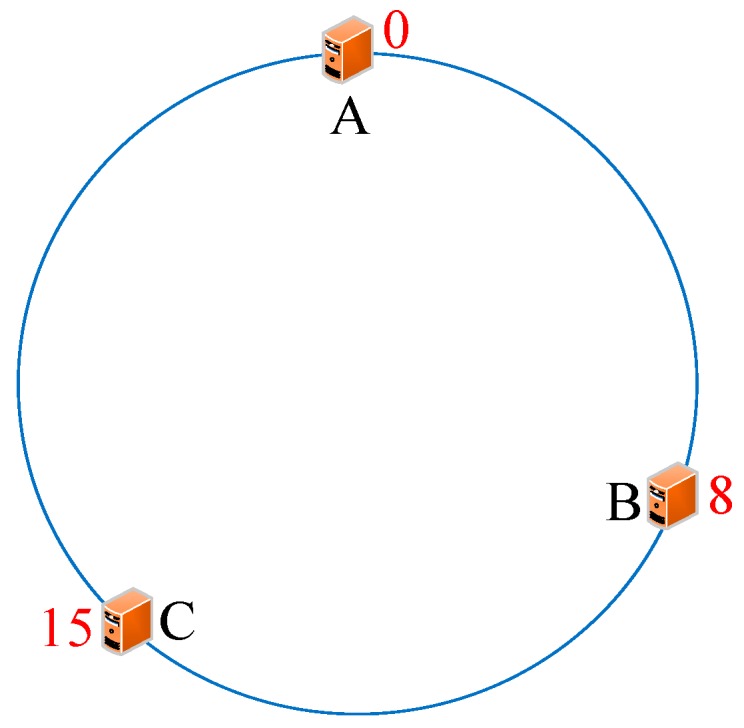
The mapping of servers to the hash circle.

**Figure 7:**
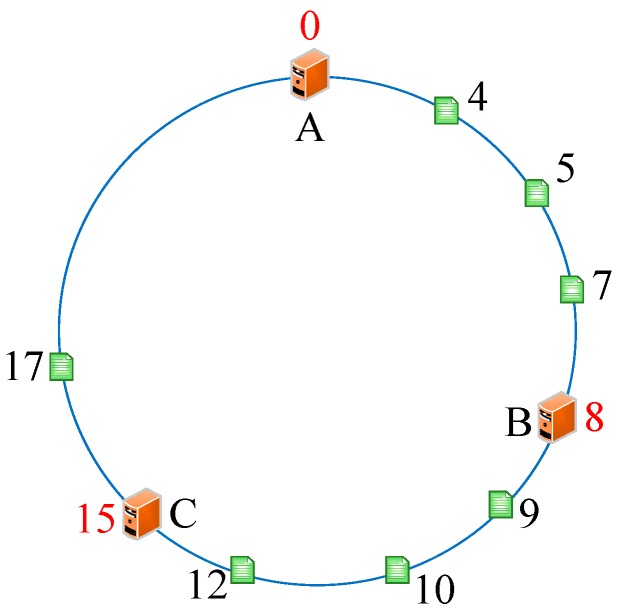
The final mapping of servers and applications to the hash circle.

**Figure 8:**
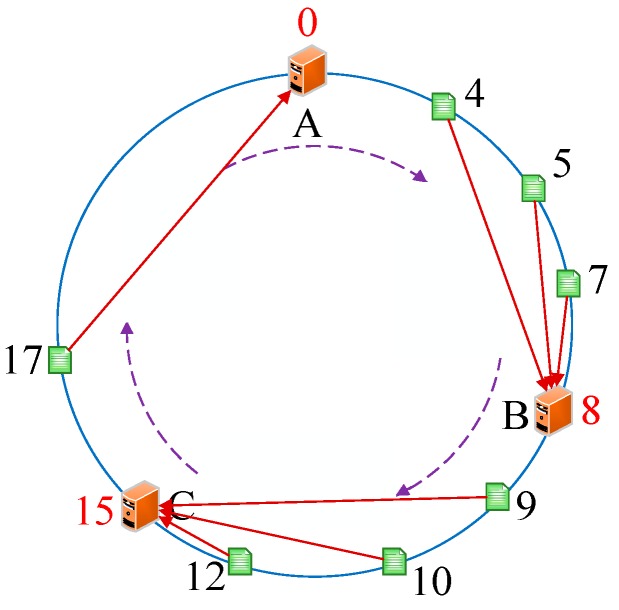
The deploying result of applications using the dynamic consistent hashing (DCH) algorithm.

**Figure 9:**
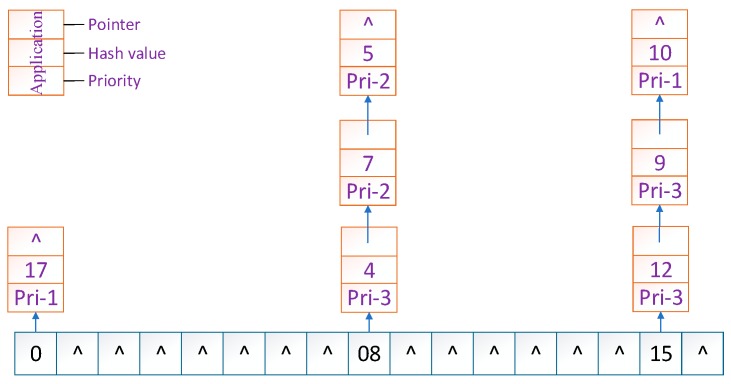
The priority-based storage structure in the DCH-based deploying algorithm.

As we know, every server has a unique IP address. Thus, we initially conducted the operations *hash (the IP address of servers)%(2^32^)* and mapped them to the hash circle according to the remainders. In order to better describe this algorithm, we assumed that there were three servers in total in the heterogeneous sensor networks, and that their remainders were 0, 8, and 15 after the hash operation. Figure 6 illustrates the mapping of servers from IP address to the hash circle. Next, we conducted the same operations *hash (name + timestamp)%(2^32^)* for seven discrete applications and mapped them to the hash circle according to the remainders. Then, we could get the remainders as a sequence, <12, 9, 4, 10, 7, 5, 17>, after the *hash ()* operation. In this way, we could get the final mapping results of servers and applications, which are illustrated in Figure 7. Finally, we deployed every application into the sever that was first encountered in clockwise direction in the hash circle. According to Figure 7, we could see that node 17 would be deployed to server A; nodes 4, 5, and 7 would be deployed to server B; and nodes 9, 10, and 12 would be deployed to server C. Then, we could get the final deploying location of the <12, 9, 4, 10, 7, 5, 17> sequence as <C, C, B, C, B, B, A>. Figure 8 depicts the deploying result of applications using our proposed DCH algorithm. Besides the load balancing, access efficiency was another issue we needed to consider. In the traditional hash-based approach, separate chaining is used to address the hash collision. Applications are stored on the same server in the form of a linked list if their remainders are the same value after the hash operation. However, this linking is based on the sequence. If we need to access or modify a target application, we have to go through every application on this server. As we know, IoT applications that require being responses in time include some emergencies, such as traffic congestion, power emergencies, or fire hazards. Thus, we designed the priority-based storage to address this issue. Applications are linked based on the value of priority. Developers only need to access those applications whose priorities match the target value, instead of going through every application on this linked list. Generally, our proposed DCH-based algorithm is a kind of hash-based algorithm. The values of the IP address for every server, as well as the name and timestamps for every application, are both random. Thus, the storage for resources in the hash circle is balanced using the DCH-based deploying algorithm. This storage structure significantly improves the access efficiency and is illustrated in Figure 9. In order to access a target resource, the following three steps are necessary: (1) Calculate the index based on the key. (2) Get the corresponding key-value pair list according to the index. (3) Find the target resource by traversing the list according to priority. Thus, the theoretical value of time complexity is O (1).

### 4.2. Deploying Algorithm for Common Cases

**Figure 10:**
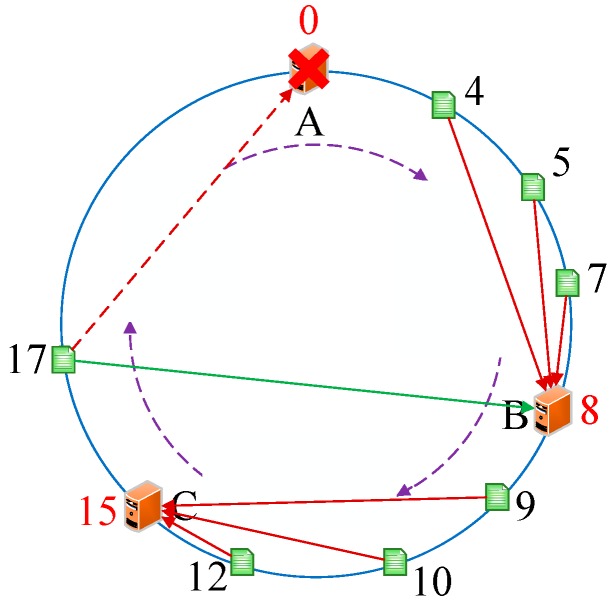
The changing of deploying locations of applications using the DCH-based algorithm.

**Figure 11:**
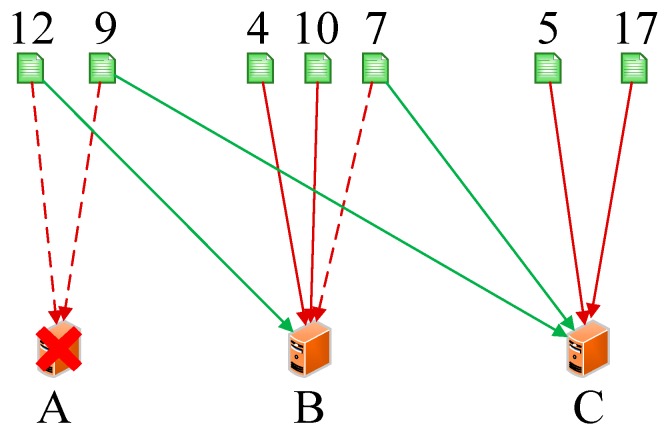
The changing of deploying locations of applications using the hash-based algorithm.

As mentioned above, we usually need to add or remove a server in an actual IoT environment. Generally, servers are discretely placed and located in different Local Area Networks (LANs). Therefore, their IP addresses are usually discontinuous and their deployed locations are not adjacent in the hash circle. Now, we analyzed the performance of the DCH-based deploying algorithm when facing the changing of server numbers. We assumed that server A becomes unavailable for some reason. According to our proposed algorithm, the mappings of server B, server C, and other applications are unchanged. Thus, only one node’s deploying location of application 17 is changed from server A to server B and the remaining nodes’ deploying locations of applications <12, 9, 4, 10, 7, 5> are unchanged. Thus, the final deploying location sequences are <C, C, B, C, B, B, B>. Figure 10 illustrates the changing of deploying locations of applications, where the green directed line points to its new deploying location. Because the sequence-based algorithm is based on a loop of the assigned constants of the servers from 1 to n, almost all applications need to be transferred when the number of available servers changes. Thus, we focus on comparing the latter two deploying algorithms. If we choose the traditional hash-based approach, the initial mapping node sequences need to be changed. We take application 17 as an example. According to the operation rule, its remainder i = 17%3 = 2, therefore, its initial deploying location is server C. Similarly, we could get the final deploying location sequence as <A, A, B, B, B, C, C>. If server A becomes unavailable, the remainder i = 17%2 = 1 and the deploying location is server C. Thus, final deploying location sequence is <B, C, B, B, C, C, C>. Figure 11 depicts the changing of deploying locations of applications using the hash-based algorithm, in which the green directed lines point to their new deploying locations. By comparison, we found that only one application’s deploying location was changed using our proposed DCH-based algorithm. However, almost half of the applications’ deploying locations were changed using the hash-based algorithm, which means that a massive cache becomes invalid and numerous applications need to be transferred when we need to access or modify a deployed application in an actual IoT system in the sensor networks, which is a resource-intensive operation in the IoT sensing environment.

### 4.3. Deploying Algorithm for Special Cases

**Figure 12:**
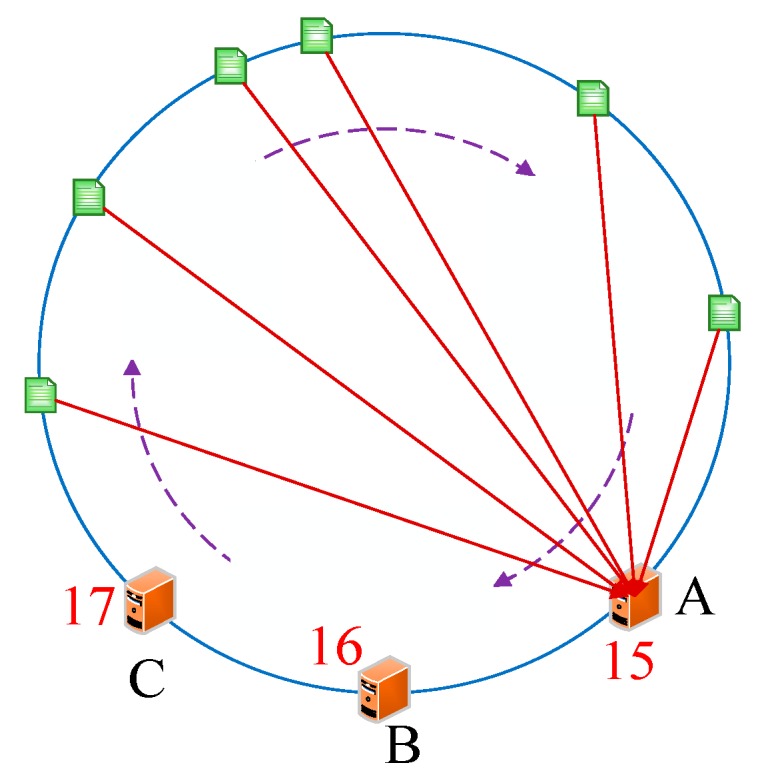
The servers are deployed in the adjacent locations in the hash circle.

**Figure 13:**
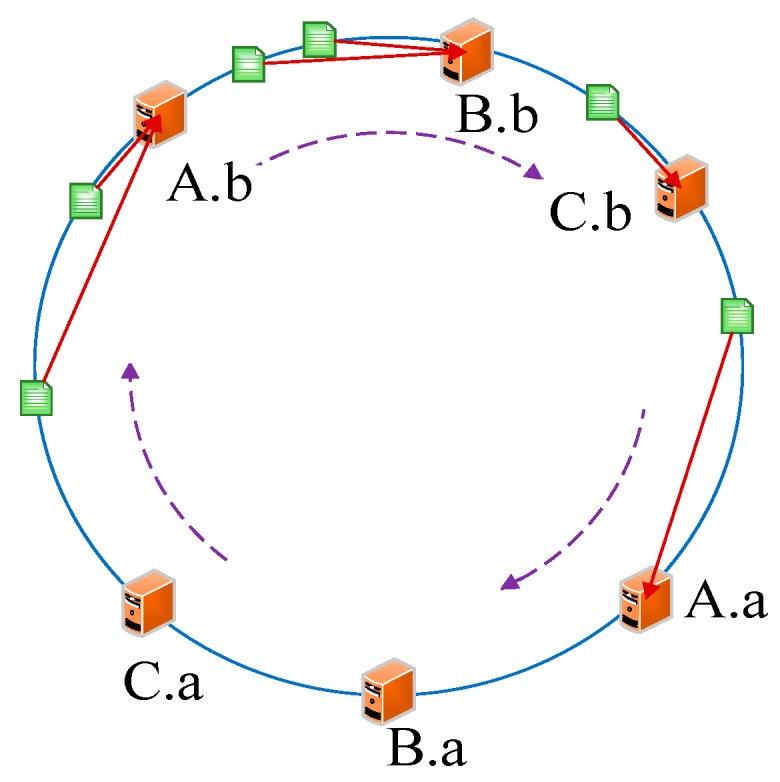
The deploying results of applications.

Servers are usually discretely placed and located in different LANs, which means that their IP addresses are usually discontinuous and their deployed locations are not adjacent in the hash circle. However, from the perspective of algorithm integrity, we should take into account the special situation in which the IP addresses of servers are adjacent. In this case, some servers will be frequently visited, meanwhile other servers are idle. To address this issue, we designed virtual nodes. A virtual node is a replica of the actual node in the hash circle. We would set two virtual nodes for an actual node in our proposed DCH-based deploying algorithm. The calculation of virtual node could adopt the IP address of the corresponding node plus digital suffix. For example, we assumed that the IP address of server X was 192.168.1.10. Without the virtual nodes, the way to calculate the hash value is *hash(192.168.1.10)*. Using the virtual nodes, the way to calculate the hash values of X.a and X.b is *hash(192.168.1.10#1)* and *hash(192.168.1.10#2)*, respectively. Algorithm 1 describes our proposed DCH-based deploying algorithm in heterogeneous sensor networks. We take the above-mentioned case as an instance, if the servers are placed at the nodes 15, 16, and 17, all applications would be deployed to server A based on the DCH-based deploying algorithm. Figure 12 illustrates the final deploying results of applications. We could see that node 15 would get the most requests because of the circular placement method. By introducing the virtual nodes, the physical node X would be turned to two virtual nodes X.a and X.b to ensure balance. The application assigned to X.a or X.b would eventually be both deployed to node X. In this way, the final server nodes placed in the hash circle are 15.a, 15.b, 16.a, 16.b, 17.a, and 17.b. As we see from the figure, the application distribution would be more decentralized. Figure 13 illustrates the deploying results of applications. 

**Algorithm 1** DCH-based deploying algorithm in the heterogeneous sensor networks.**Input:** The IP address queue of servers Q_1_ = (IP_1_, IP_2_, …, IP_n_) and the naming queue of applications Q_2_ = (AP_1_, AP_2_, …, AP_n_)**Output:** The deploying results of the applications Q_3_ Conduct an hash circle that consisted of 2^32 nodes **for** Q_1_ is not empty **do**   **If** (the elements in Q1 are adjacent) **then**   Conduct the operation i = *hash (IP)%(2^32)* for every element in Q_1_   **else**     Conduct the virtual node X.a = *hash (IP#1)%(2^32)* for node X     Conduct the virtual node X.b = *hash (IP#2)%(2^32)* for node X   Map the server to hash circle according to the remainder i **for** Q_2_ is not empty **do**   Conduct the operation j = *hash (name+timestamp)%(2^32)* for every element   Map the application to hash circle according to the remainder j Access the nodes of applications in clockwise direction in hash circle **if** (!hash circle.containsKey ()) **then**   Deploy the application to the current server   **if** (newNode.getPriority() > currentNode.getPriority()) **then**     Link the new node before the current node   **else** Link the new node after the current node**else** Deploy the application to the server 0**return** The deploying results of the applications Q_3_

## 5. Experiment Results

We have qualitatively and quantitatively analyzed the DCH-based deploying algorithm with another two algorithms based on a limited data set in the above section. The logic and operation of the sequence-based algorithm is the simplest. All servers are assigned constants from 1 to n at the initialization time. Users deploy applications based on a loop of the assigned constants. However, its shortcoming is lack of flexibility. If we need to access or modify a deployed application, we have to go through all applications on the servers. Ideally, we could query the target applications at once without any comparison. Then, it is necessary to establish a definite correspondence *f* between the storage location of the application and its keywords. When searching, the target value *f(keywords)* could be found only according to the correspondence relationship. The traditional hash-based algorithm is just an algorithm based on this method. Therefore, we would choose these two algorithms for performance comparison in this section. To further evaluate these algorithms, we conducted a series of simulation experiments on the basis of larger data sets.

**Figure 14:**
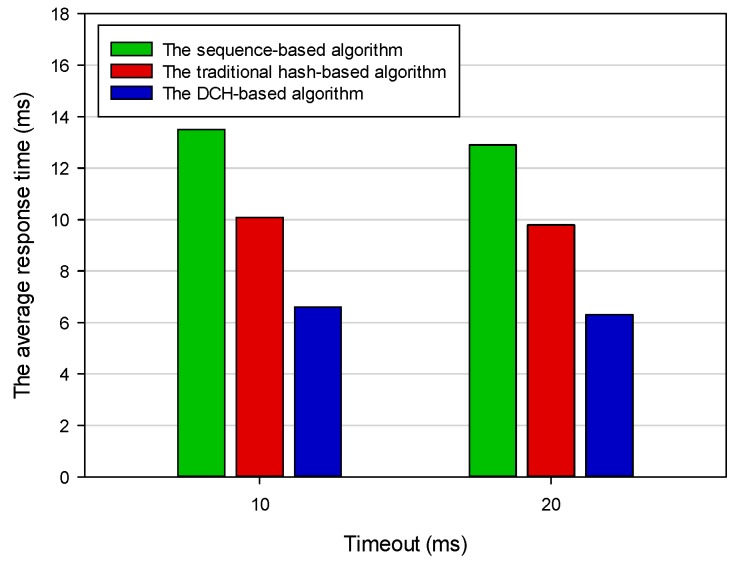
The average response time based on different deploying algorithms.

**Figure 15:**
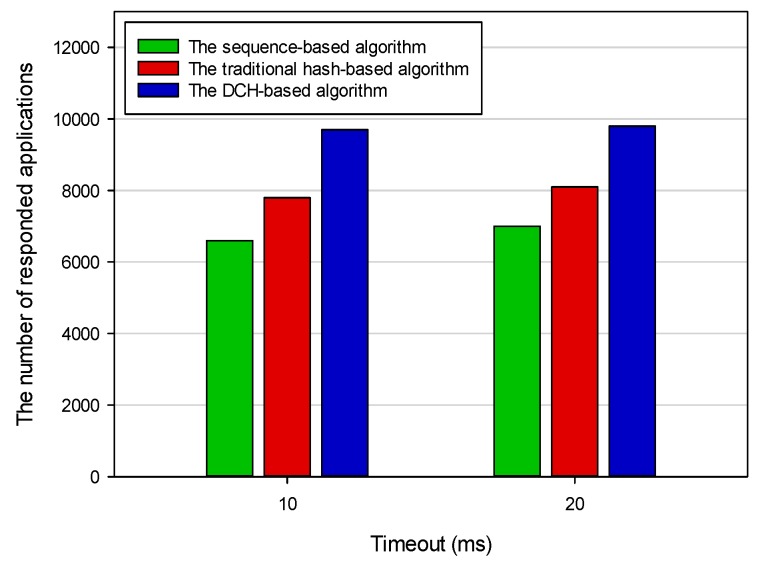
The throughput based on different deploying algorithms.

The simulation experiments were conducted on four of the same PCs, which had 6 GB of RAM, 3.07 GHz CPU, and 500 GB of disk space. One PC (we named it A) was simulated to deploy the designed applications or access the deployed applications and the other three PCs (we named them B, C, and D) were simulated as servers to store these applications. We allocated 1 GB of RAM and 2 GB of disk space of every PC for the following experiments. In order to simulate the raw data gathered by sensors and applications developed by users in the IoT execution environment, PC A sent ten data packets to the unified message space every 1 millisecond. In addition, we randomly specified the data size and the corresponding priority of the data packet. The data size ranged from 10 k to 500 k and the initial priority ranged from 1 to 5. To get closer to reality, we designed a threshold timeout. All applications whose response time exceeded the threshold would be abandoned. We repeatedly conducted the experiments under the condition that the values of timeout were set to 10 ms and 20 ms. The sampling time was 1000 ms and the total number of deployed applications was 10 thousand. The final results were illustrated as follows:

Response time was the total amount of time taken by the IoT system to respond to a request for execution. Ignoring transmission time for a moment, the response time was the sum of waiting time and execution time. Figure 14 shows the average response time based on different deploying algorithms. The average response times of the sequence-based algorithm, the DCH-based algorithm, and the traditional hash-based algorithm were 12.876 ms, 6.925 ms, and 10.028 ms, respectively. The response time in the DCH-based algorithm was 46 percent and 31 percent less than the other two algorithms, respectively. In the sequence-based algorithm, all applications were evenly deployed to three servers. When we required access to a deployed application, we had to go through all the deployed applications of the three servers. The time complexity of this algorithm was *O (n)*. In the traditional hash-based algorithm, we could get the determined server address that stored the target application according to hash operation rule. However, we still had to go through all the deployed applications on this server. In our proposed DCH-based algorithm, the priority was used as the secondary index to query the target application. The datasets that needed to be processed in this algorithm were only a subset of the traditional hash-based algorithm. Thus, the average response time of querying a deployed application in the DCH-based algorithm was the shortest. Throughput was the total number of applications that had been responded to by the IoT system within a unit of time. Figure 15 shows the final results of throughput based on different deploying algorithms in the heterogeneous execution environment. The average throughput of the sequence-based algorithm, the DCH-based algorithm, and the traditional hash-based algorithm was 6976, 9728, and 8012, respectively. The throughput in the DCH-based algorithm was 39 percent and 21 percent more than the other two algorithms, respectively. The value of throughput was directly affected by the average response time. As mentioned above, the average response time in the DCH-based algorithm was the shortest. Thus, it could respond to more applications than the sequence-based and traditional hash-based algorithms.

**Figure 16:**
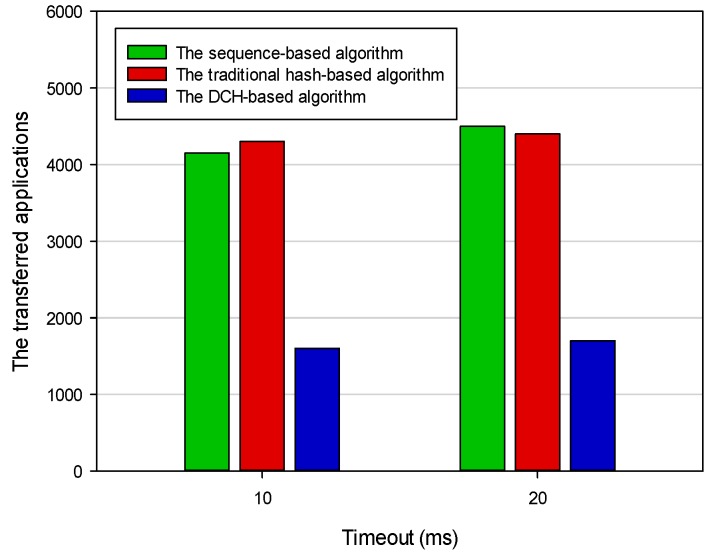
The transferred applications based on different deploying algorithms.

**Figure 17:**
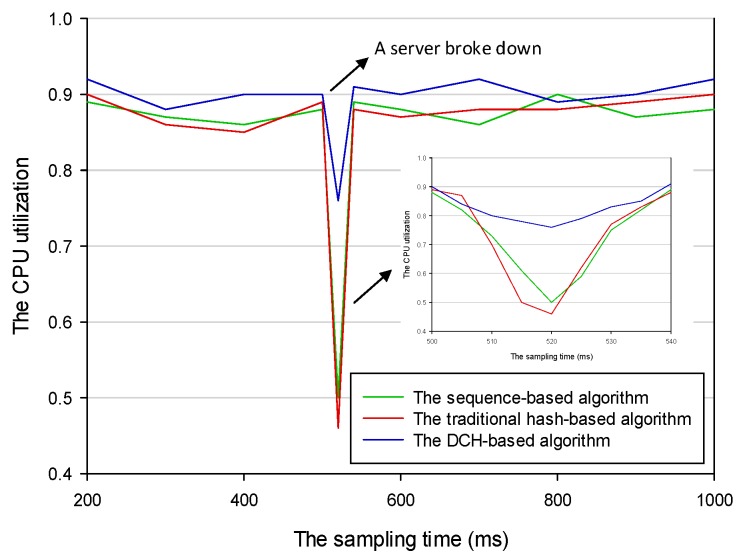
The CPU utilization of the Internet of Things (IoT) system based on different deploying algorithms.

To simulate the scene in which a server became unavailable when the status of the IoT system was running, we shut down one PC after it had run for 500 ms. We repeatedly deployed those allocated applications to the remaining two servers according to three different deploying algorithms. By comparing the results of the two experiments, we found that the number of applications transferred in the DCH-based algorithm was the least. In this case, only those applications stored on the server that was shut down needed to be transferred. However, almost all applications stored on the three servers needed to be fully redeployed using the sequence-based and the traditional hash-based algorithms. Figure 16 shows the results of applications that needed to be transferred if a server became unavailable based on the three different deploying algorithms. The number of transferred applications of the sequence-based algorithm, the DCH-based algorithm, and the traditional hash-based algorithm was 4318, 1602, and 4256, respectively. The average number of transferred applications in the DCH-based algorithm was 63 percent and 62 percent less than the other two algorithms, respectively. In order to improve the system utilization, we should try our best to keep the CPU busy and reduce the time consumption of the I/O operation. Figure 17 shows the CPU utilization of the IoT system with different application deploying algorithms. We intercepted the results of the period from 500 ms to 540 ms. The results showed that the thrashing in the DCH-based algorithm was the slightest compared with the sequence-based and the traditional hash-based algorithms when a running server suddenly became unavailable. Because the transferred dataset in the DCH-based algorithm was only a subset of the other two algorithms, it could better adapt to the dynamic IoT execution environment.

## 6. Implementation and Discussion

On the basis of the above work, we extended the BPMN plugin in Eclipse and developed a series of custom functions to achieve our goals. In this section, we will describe the implementation in detail and compare our deigned system with other systems. Furthermore, we will discuss the advantages and disadvantages of our designed system with a practical example.

**Figure 18:**
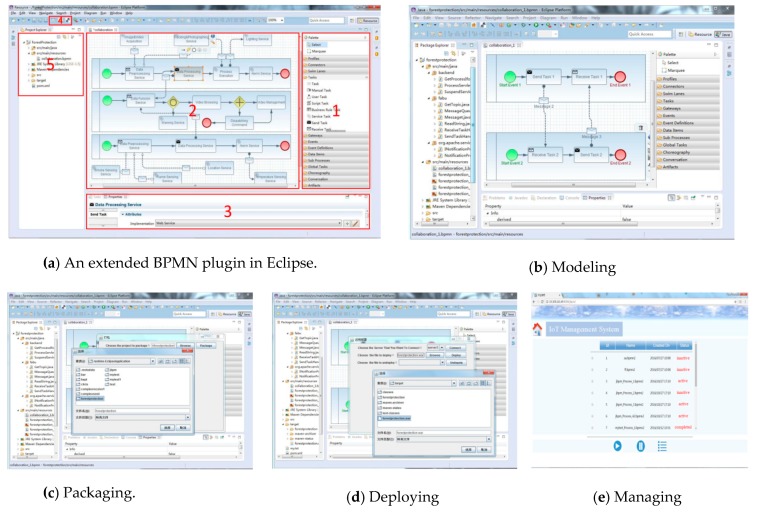
These are screenshots of our designed platform. (**a**) An extended BPMN plugin; (**b**) modeling the business process application; (**c**) packaging the application into a war file; (**d**) deploying the war file to discrete servers; and (**e**) managing the deployed applications.

Figure 18a illustrates our extended BPMN plugin. It consists of five major parts and provides all essential tools for business process developers. Each part is responsible for a different function. As mentioned in Section 3, we integrated the paradigm of sensor devices as a business process resource that could be directly represented in the basic process element library. Part 1 illustrates this basic element library, which provides all kinds of draggable business process elements (e.g., tasks, events, gateways, etc.). Developers could directly model a sensor-aware business process application in this way. As a tool to model discrete IoT systems, Petri nets have been widely used to model the business process applications [32,33]. In another paper of ours [27], we have extended the traditional Petri nets with a sensing factor, allowing it to support the modeling of collaborative business processes towards IoT applications. Using that approach, a sensor task could be formally described by a transition that has one input place and one output place, representing the start and end of the sensor task, respectively. Besides, because of the importance of sensor data, their corresponding places are added into the extended Petri nets model. Part 2 is a workspace panel where developers model the specific business process, creating the corresponding extended Petri nets and binding the sensing data to the sensor task. As we say, the business process in a cross-sensor application is usually collaborative. Thus, we encapsulate raw sensor data into a unified message format and forward them through a send/receive task, because sensor devices are the source of data and provide different kinds of services. Figure 18b shows a designed collaborative business process. We developed a configuration panel in Part 3 for developers to configure the properties of the process elements, including the interface, timeout, and application priority. Valid values are “##unspecified” for leaving the interface technology open, “##Restful” for the restful technology, or a URL identification and other technology or coordination protocol. Because of the widespread application of restful technology in the sensor networks, restful is the default technology. Besides modeling, how to efficiently deploy a designed application is another pressing issue. Three custom buttons are designed in Part 4 for developers to decouple the business processes into fine-grained process fragments, package the IoT application into a war file, and deploy the war file to distributed servers. In order to support a failure-transparent execution of servers, our platform gathers the periodic heartbeat to show their liveness. Whenever a server fails to send the heartbeat message after a given timeout, our platform would mark it as unavailable to avoid invalid deploying. Figure 18c,d show the way to package and deploy applications. Our proposed DCH-based deploying algorithm is used in this phase. Part 5 is a package explorer, using which developers could edit the element hierarchy of the IoT-aware business process applications. As we know, the status of a business process instance includes *NOTSTARTED*, *RUNNING*, *SUSPEND*, and *CANCEL*. Thus, we also support to manage deployed applications. Developers could start up or suspend them by clicking on the appropriate buttons in the bottom of Figure 18e, as well as examine their information, including id, name, created time, and running status.

As mentioned above, our developed work is based on an open source project JBPM 6.1.0, which is an Eclipse-based editor to support the graphical creation of business process applications. Besides this, Activiti [34] is also a leading lightweight, Eclipse-based BPMN instrument supporting business process automation needs. Compared with these, the differences and advantages of our system are as follows: (1) These two existing instruments could not support the direct modeling of sensor-associated business process applications. When introducing sensor services, developers have to deal with amounts of auxiliary operations. For example, a special *Service Task* is provided for developers to access to external services in JBPM 6.1.0. However, it only gives the abstract class and requires developers to implement complex logic themselves. If using Activiti, its execution engine could not directly parse the sensor-associated service, which is not known in XML format to the BPM environment. In contrast to these, we integrated sensor services into a particular *Sensor Task* in our system. Developers could directly model a machine-readable sensor-aware business process without any extra operations. (2) On the basis of existing functions of JBPM 6.1.0 and Activiti, when deploying designed business process applications to servers, developers have to manually enter complex deployment commands in the console, which not only takes a lot of time, but also tends to introduce mistakes. Besides, developers have to personally specify the target servers to be deployed for applications, lacking the consideration of load balancing. In our system, we integrated deployment commands and the DCH-based deploying algorithm into a custom button. Developers could complete the deployment by clicking on the appropriate button. Although we have done most of the work, as far as our present work was concerned, the applications on invalid servers have to be redeployed to remaining servers again based on the DCH-based deploying algorithm. In order to address this disadvantage, we considered that applications might be automatically transferred to adjacent available servers based on some rules at the lowest cost, instead of recalculating by our system in a resource-limited environment. Thus, the optimization strategy of the application transferring approach will be another research focus in our future work.

## 7. Case Study

**Figure 19:**
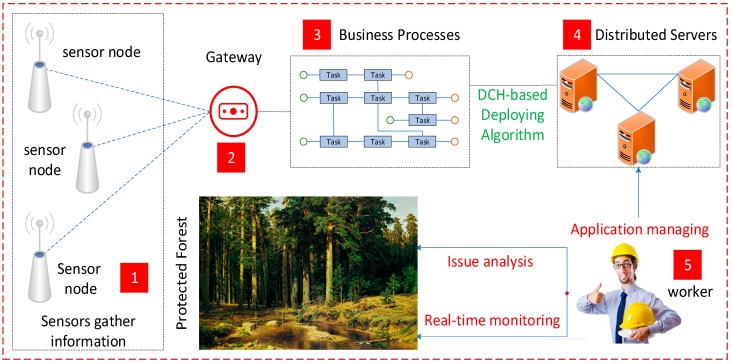
An actual sensor-aware business process application using our designed system

We have developed an actual sensor-aware business process application using our designed system to protect a large area of forest and applied it in North China. The work of forest-protection used to be done by the way of human mountaineering in the past. However, this way not only costs a great deal of resources, but also could not deal with emergencies in time. Using our designed IoT system, numerous smart sensors are discretely deployed in multiple monitoring areas. These smart sensors are in charge of collecting their surrounding information and sending the corresponding data to the cloud for further processing and analyzing. Because of the heterogeneity of sensing data, the system would encapsulate them before they are submitted to the unified messaging space. In general, this forest-protection application usually involves the following sensors: the fire sensor, the infrared thermal imaging sensor, and the tracking camera sensor. Figure 19 illustrates this business process application using our designed system. The scenario includes the following steps:

The fire sensor collects its surrounding information and regularly sends these raw data to the smart gateway for further filtering, classifying, and aggregating.

When an emergency occurs in the monitoring areas, the fire sensor publishes a fire event.

Distributed servers route this fire event and publish a command event to the infrared thermal imaging sensor, making it detect whether there are moving targets (e.g., explorers, campers, etc.).

If they are detected, the infrared thermal imaging sensor publishes a detected event in time.

Distributed servers receive this event and start up the tracking camera sensor.

The tracking camera sensor keeps track of the moving targets, transmits the actual information, and requires entity resources from the remote dispatch center.

As we see, this application involves three kinds of sensors. They have their own functions and interact with each other to accomplish forest protection. In a traditional IoT system, we have to separately create a corresponding business process model for a sensor device. As we say in the previous section, if introducing sensor services, we have to conduct many auxiliary operations. In our system, we could directly model this sensor-aware business process application at once. Besides the above-mentioned sensor devices, there are other types of smart sensors that are also usually applied in the forest, such as the temperature senor, the humidity sensor, and the carbon dioxide sensor. All these sensor-aware business process applications are deployed and stored on discrete servers. The traditional IoT system leaves all deployment work to developers and lacks appropriate deploying strategies. Thus, some servers might be required the most while other servers are idle, which could not ensure load balancing in a resource-constrained environment. In addition, we usually need to dynamically add or remove servers in the forest protection scenario, which means that a lot of applications have to be transferred and redeployed. In contrast to the traditional IoT system, our system deploys applications based on the DCH-based algorithm to ensure load balancing. If the number of servers change, only a part of applications need to be redeployed. Furthermore, we assign priority to each application in this scenario. Applications are linked based on the priority value. If we tend to modify a deployed application, we only need to access those applications whose priorities match the target value, instead of going through every application on the linked list. On this basis, the overall performance has been significantly improved.

## 8. Conclusions

This paper implemented direct modeling for an IoT-aware business process application and ensured load-balanced deploying using the DCH -based deploying algorithm. To reach this goal, we initially extended the graphic model and machine-readable model specification of BPMN 2.0, allowing it to support the direct modeling of a sensor device. Then, we proposed a DCH-based deploying algorithm to solve the problem of dynamic load balancing and access efficiency in the distributed execution environment. Finally, we analyzed the feasibility of our proposed approach and designed an actual IoT-aware business process application in the forest-protection scene. Generally, with more applications used, the throughput in the DCH-based algorithm was the largest. Because the priority was used as the secondary index to query the target application, we only needed to go through a subset of the whole datasets. If a running server suddenly became unavailable, only those applications stored on this server needed to be transferred in the DCH-based algorithm, instead of redeploying all applications using the sequence-based or traditional hash-based algorithms. Thus, the thrashing in the DCH-based algorithm was the slightest compared with the other two algorithms when failure occurred, and the DCH-based algorithm could better adapt to the dynamic IoT execution environment. As far as our present work was concerned, the applications on invalid servers had to be redeployed to remaining servers again based on the designed deploying algorithm. However, the optimization strategy of the application transferring approach will be another research focus in our future work. We considered that applications might be automatically transferred to adjacent available servers based on some rules at the lowest cost, instead of recalculating using the IoT system in a resource-limited environment.

## Figures and Tables

**Table 1:** Interface attributes and model associations.

Attribute Name	Description
name: string	The descriptive name of the element.
operations: Operation [1, ..., *]	The operations that define the Interface. An Interface has at least one Operation.
callableelement: Callableelement [0, ..., *]	The CallableElements that use this Interface.
implementationRef: Element [0, ..., 1]	This attribute allows to reference a concrete artifact in the underlying implementation technology representing that interface.

**Table 2:** Interface XML schema definition (XSD) specification.

<xsd: element name = “interface” type = “Interface” Group = “baseElement”/>
<xsd: complexType name = “tInterface”>
<xsd: complexContent>
<xsd: extension base = “tBaseElement”>
<xsd: sequence>
<xsd: element ref = “operation” minOccurs = “1” maxOccurs = “10”/>
</xsd: sequence>
<xsd: attribute name = “Qname” type = “xsd: string” use = “required”/>
<xsd: attribute name = “Qname” type = “xsd: string” use = “optional”/>
</xsd: extension>
</xsd: complexContent>
</xsd: complexType>
